# Lesion of the Olfactory Epithelium Accelerates Prion Neuroinvasion and Disease Onset when Prion Replication Is Restricted to Neurons

**DOI:** 10.1371/journal.pone.0119863

**Published:** 2015-03-30

**Authors:** Jenna Crowell, James A. Wiley, Richard A. Bessen

**Affiliations:** 1 The Prion Research Center, Department of Microbiology, Immunology, and Pathology, Colorado State University, Fort Collins, Colorado, United States of America; 2 Department of Microbiology and Immunology, Montana State University, Bozeman, Montana, United States of America; University of Verona, ITALY

## Abstract

Natural prion diseases of ruminants are moderately contagious and while the gastrointestinal tract is the primary site of prion agent entry, other mucosae may be entry sites in a subset of infections. In the current study we examined prion neuroinvasion and disease induction following disruption of the olfactory epithelium in the nasal mucosa since this site contains environmentally exposed olfactory sensory neurons that project directly into the central nervous system. Here we provide evidence for accelerated prion neuroinvasion and clinical onset from the olfactory mucosa after disruption and regeneration of the olfactory epithelium and when prion replication is restricted to neurons. In transgenic mice with neuron restricted replication of prions, there was a reduction in survival when the olfactory epithelium was disrupted prior to intranasal inoculation and there was >25% decrease in the prion incubation period. In a second model, the neurotropic DY strain of transmissible mink encephalopathy was not pathogenic in hamsters by the nasal route, but 50% of animals exhibited brain infection and/or disease when the olfactory epithelium was disrupted prior to intranasal inoculation. A time course analysis of prion deposition in the brain following loss of the olfactory epithelium in models of neuron-restricted prion replication suggests that neuroinvasion from the olfactory mucosa is via the olfactory nerve or brain stem associated cranial nerves. We propose that induction of neurogenesis after damage to the olfactory epithelium can lead to prion infection of immature olfactory sensory neurons and accelerate prion spread to the brain.

## Introduction

Prion diseases can have an infectious, genetic, or sporadic etiology, but the most common origin among ruminants such as sheep and cervids who contract scrapie and chronic wasting disease (CWD), respectively, is by infection. Based on the distribution of the misfolded, disease-associated prion protein (PrP^Sc^) during the early stages of natural infection it has been deduced that prion infection is orally acquired [1–4]. Early PrP^Sc^ deposition in the lymphoreticular and peripheral nervous systems of the gastrointestinal tract, and subsequent spread to the central nervous system has been experimentally confirmed following oral exposure to prions [5–7]. A central event in transmission of prion diseases is the establishment of infection in the lymphoreticular system (LRS), which is the primary site for prion replication following peripheral infection. After prion exposure, infection is typically established in the draining lymph node prior to dissemination throughout the LRS and entry into peripheral nerves, which serve as a conduit for prion spread to the central nervous system [8–10]. Blood borne prion infection may also lead to entry into the CNS is some cases [10]. Due to the widespread distribution of prions in the LRS the role of mucosae, other than the gastrointestinal tract, as a site of prion entry in natural infection has neither been confirmed nor disproven.

Experimental studies indicate that additional mucosae can serve as sites of prion entry. Application of prions to several mucosal surfaces, especially those with a high density of innervation, can result in neuroinvasion independent of LRS infection [11,12]. For example, immune deficient mice with immature follicular dendritic cells, which cannot replicate prions in the LRS, are susceptible to prion aerosols and intranasal inoculation of RML scrapie [11,13]. In other cases, Syrian hamsters are not susceptible to DY TME infection, a neurotropic strain that does not replicate in the LRS, by the intranasal route. However, hamsters are susceptible to HY TME infection, which is a related prion strain that is both lymphotropic and neurotropic [12]. In ruminants, physical disruption or microbial infection of mucosae is a common event and has been proposed to enhance prion uptake and transmission [14]. For example, several studies demonstrate that an experimental lesion that disrupts the integrity of the lingual mucosa can result in a reduction in the prion incubation period and an increase in disease penetrance [14,15].

In the current study we investigated prion neuroinvasion from the olfactory epithelium since this mucosa contains environmentally exposed neurons that are susceptible to prion infection, it has been implicated as a site for prion entry, and is susceptible to damage by environmental, chemical, microbial, and inflammatory insults [16–24]. An olfactory toxin was used to induce apoptosis in olfactory sensory neurons and a transient loss of the olfactory epithelium, which stimulates neurogenesis and regeneration of neurons and the olfactory epithelium [25,26]. Following damage to the olfactory epithelium, intranasal prion inoculation resulted in a shortening of the incubation period and/or an increase in disease penetrance in rodent models with neuron-restricted prion replication. Analysis of early brain infection in the neuron-restricted models indicated initial prion entry into either the brain stem or olfactory bulb in the nasotoxic lesion group, but no evidence of progression of early brain infection in the control group. These findings suggest that disruption of the olfactory epithelium and/or regeneration of olfactory sensory neurons can enhance prion infection of olfactory mucosa- and/or brain stem-associated cranial nerves, and accelerate the course of prion infection and onset of disease.

## Materials and Methods

### Animal inoculations and tissue collection

All procedures involving animals were approved by the Colorado State University IACUC and were in compliance with the *Guide for the Care and Use of Laboratory Animals*; these guidelines were established by the Institute of Laboratory Animal Resources and approved by the Governing Board of the U.S. National Research Council. Two days prior to inoculation of prions, disruption of the olfactory epithelium was performed by injection of vehicle or methimazole (Sigma-Aldrich Co., St. Louis, Mo.) into the peritoneum at 100 mg/kg for mice and 125 mg/kg for Syrian golden hamsters in phosphate buffered saline containing 10% dimethyl sulfoxide. Weanling, outbred Syrian golden hamsters (Simonsen Laboratories, Gilroy, CA) were inoculated with several 10-fold dilutions of a brain homogenate from a normal hamster (i.e., mock infected group), a HY TME infected hamster containing 10^9.5^ intracerebral lethal median dose (LD_50_) per gram, or a DY TME infected hamster containing 10^7.5^ intracerebral LD_50_ per gram by the intranasal (20 μl) and intracerebral (50 μl) routes as previously described [12]. Both C57Bl/6J mice (Jackson Laboratories, Bar Harbor, ME) and HPrP7752KO transgenic mice expressing the Syrian golden hamster prion protein gene (*Prnp*) controlled by the rat neuron enolase promoter on a *Prnp* null background (gift from Bruce Chesebro, NIH Rocky Mountain Laboratories, Hamilton, MT, USA)[27,28] were inoculated by the intranasal and intracerebral routes with a brain homogenate from either RML scrapie (10^7.5^ intracerebral LD_50_ per gram) infected mice or HY TME infected hamsters. Following prion inoculation rodents were observed three times per week for the onset of neurological disease as previously described [11,12,29]. Rodents were euthanized at the early stages of clinical disease or at select time points postinoculation. Following euthanasia, tissues were collected and frozen for Western blot analysis, or the animals were perfused with periodate-lysine-paraformaldehyde (PLP), tissues dissected, and processed for embedding in paraffin wax as previously described [14,30].

#### Immune cell counts

Nasal lavages were collected from vehicle and methimazole groups prior to treatment and on days 2, 5, 7 and 10 following treatment. Hamsters were anesthetized with isofluorane and one ml of PBS in a syringe with a blunt needle tip was slowly dispensed into the left nostril. The nasal lavage was collect from the right nostril after the PBS rinsed through the sinuses. The number of viable cells in nasal lavages was determined following exclusion of trypan blue and cell counting using a hemacytometer. Cell differential counts were performed using cytospin chambers and Diff-Quik cell staining (Siemens Healthcare Diagnostics, Newark, NJ) in order to determine the number of neutrophils, macrophages, lymphocytes, and eosinophils in nasal lavages at each time point. In most samples a minimum of 3,000 cells were counted.

### PrP^Sc^ enrichment and western blot

Frozen brain, olfactory bulb and nasal turbinate were homogenized in lysis buffer (i.e., 10 mM Tris-HCL, pH 7.4, 150 mM NaCl, 1 mM EDTA, 0.5% sodium deoxycholate and 0.5% ipegal) containing 1X complete protease inhibitor (Roche Diagnostics, Indianapolis, IN) to approximately 10% (weight per volume). Tissues were homogenized in a Bullet Blender (Next Advance, Averill Park, NY) using either glass (brain and olfactory bulb) or zirconium (nasal turbinate) beads. The protein concentration in tissue homogenates was determined using the micro-BCA assay (Pierce Protein Research, Rockford, IL). For detection of PrP^Sc^ in brain and olfactory bulb homogenates, protein (100 to 300 μg) from tissues of clinically ill rodents was digested with proteinase K (Roche Diagnostics Corporation, Indianapolis, IN) at 10 μg/ml at a protein sample concentration of 1 mg/ml and incubated at 37°C for one hour with constant agitation followed by the addition of 1 mM PefaBloc (Roche Diagnostics Corporation, Indianapolis, IN). To enrich for PrP^Sc^ in tissue from rodents during preclinical disease, tissue (<10 mg to 200 mg) was extracted with detergent (10% [wt. vol.] N-lauroylsarcosine in 10 mM Tris-HCl, [pH 7.5], 100 mM NaCl, 1 mM EDTA, and 1 mM dithiothreitol) and subject to ultracentrifugation prior to proteinase K digestion as previously described [11].

For the time course study, at each week postinoculation the brain was removed, cut along the midline, and half the brain was frozen (and further dissected into olfactory bulb and brain stem) and half was placed in PLP fixative. For western blot analysis on frozen tissue PrP^Sc^ was enriched from brain homogenates by extraction in buffer containing 10% sarkcosyl, differential ultracentrifugation, and proteinase K digestion. The following tissue equivalents were used for PrP^Sc^ analysis, ≤10 mg of olfactory bulb, 10 mg of brain (minus olfactory bulb and brain stem) from week 10 through week 12 of methimazole treated animals, and 20 mg of brain and brain stem from all vehicle treated animals, and brain and brain stem samples from weeks 8 and 9 following methimazole treatment.

SDS-PAGE and western blot analysis was performed in 12% MOPS NuPAGE gels (Invitrogen, Carlsbad, CA) and transferred to PVDF membranes as previously described [31]. For detection of prion protein immunodetection was performed with murine anti-D13 PrP monoclonal antibody as previously described [32].

### Immunohistochemistry

Skulls containing the nasal cavity and olfactory bulb were collected after PLP fixation, decalcified, embedded in paraffin wax and immunohistochemistry (IHC) for PrP^Sc^ and olfactory marker protein (OMP) was performed as previously described with modifications [14]. Briefly, animals were intracardially perfused with PLP fixative followed by immersion fixation of tissues in PLP for an additional five to seven hours, or overnight for skulls. Alternatively, half the brain was immersion fixed in PLP for 24 hours after it was cut along the midline in the sagittal plane. Tissue sections were subjected to antigen retrieval by treatment with formic acid (99% wt. vol.) for 30 minutes. This was followed by successive incubation with anti-PrP monoclonal 3F4 antibody [33] or anti-OMP polyclonal antibody (Wako Chemicals, Richmond, VA) for overnight at 4°C, horse anti-mouse IgG conjugated to biotin (1:400) or donkey anti-goat conjugated to horseradish peroxidase (HRP)(1:2,000) at room temperature for 30 minutes, and streptavidin-HRP at room temperature for 20 minutes. PrP^Sc^ and OMP were visualized by localization of HRP activity with DAB+ (Dako, Carpinteria, CA) as previously described [12,31]. Tissue sections were counterstained with hematoxylin and coverslip mounted with Aquamount (Lerner Laboratories, Pittsburgh, PA) for viewing with a Nikon Eclipse E600 microscope. Controls for PrP^Sc^ IHC included the use of mock-infected tissues and substituting a similar concentration of murine IgG isotype control for the anti-PrP 3F4 monoclonal antibody.

### Statistical Analysis

Prism software (Graphpad, www.graphpad.com) was used to perform statistical analyses including two-way ANOVA and Bonferroni tests, and Mantel-Cox log-rank test. Values of P<0.05 were considered to be statistically different.

## Results

### Olfactory toxin induces inflammation, loss of olfactory epithelium, and regeneration of olfactory epithelium

**Fig 1:**
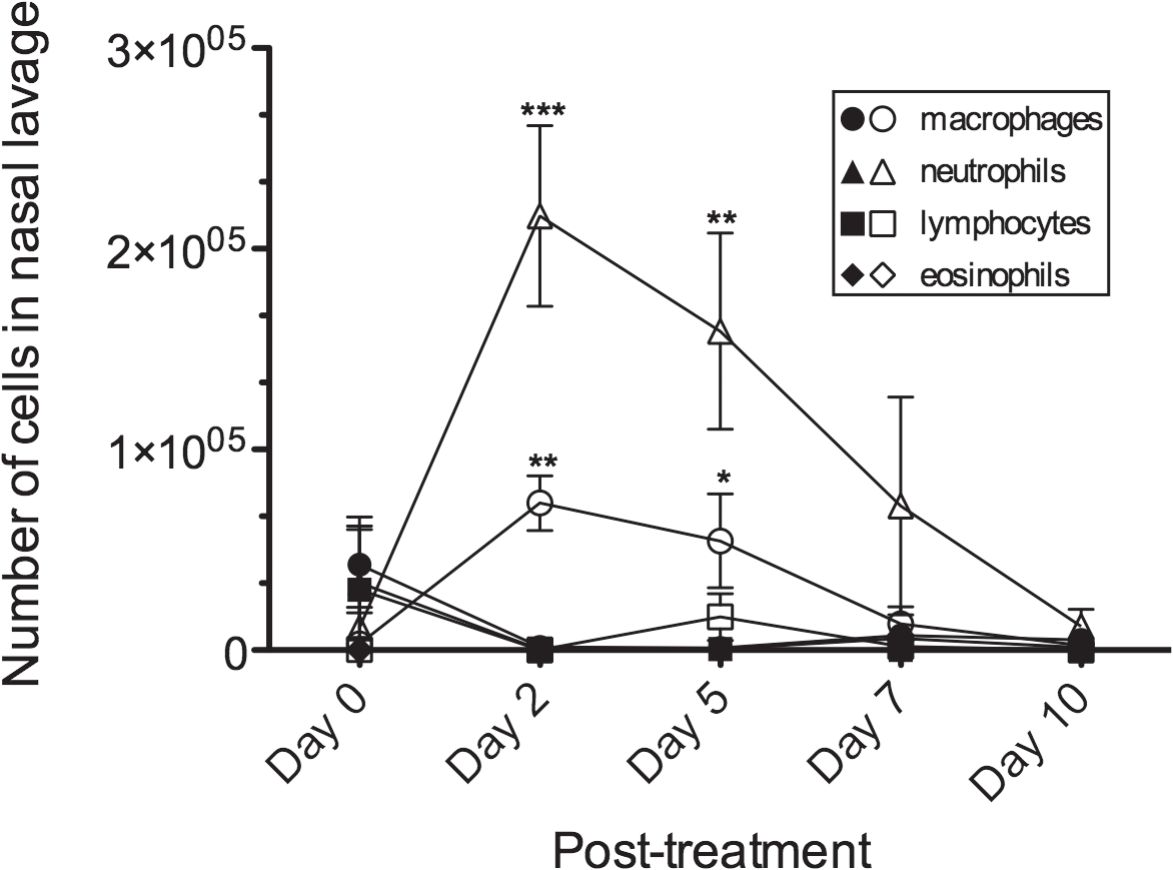
Nasotoxic treatment causes infiltration of neutrophils and macrophages into the nasal airway. Syrian golden hamsters were injected into the peritoneum with vehicle (closed symbols) and methimazole (open symbols) and nasal lavages were collected in PBS prior to (at day 0) and after treatment. Two-way ANOVA was used to statistically compare curves between vehicle and methamizole treatment groups (N = 3 hamsters) and, if a difference was found, a Bonferroni post-test was used to compare the number of cells at each collection point (*, P < 0.05; **, P < 0.01; ***, P < 0.001).

To investigate the effect of disruption of the olfactory epithelium (OE) on the susceptibility to experimental prion disease, Syrian golden hamsters were treated with either vehicle or methimazole two days prior to intranasal inoculation of prions. Methimazole is an antithyroid drug used to treat hyperthyroidism and has several side effects including a loss of taste and smell [34,35]. In rodents methimazole is used as an olfactory toxin; it is an inhibitor of cytochrome P450 in sustenacular cells located in the OE and duct cells of the Bowman’s gland in the lamina propria of the olfactory mucosa [36]. Methimazole-induced necrosis of these cells leads to apoptosis of olfactory sensory neurons (OSNs) and sloughing off of the OE into the nasal airway [31,37–39]. Since damage to the OE is likely to elicit an innate immune response, which may affect early prion pathogenesis, we measured the number and types of infiltrating immune cells that were present in the nasal airways after vehicle and methimazole treatment. Nasal lavages were collected prior to treatment and at days 2, 5, 7, and 10 after treatment (Fig. 1). A comparison between treatment groups indicates that there was a statistical difference in neutrophils and macrophages, but not lymphocytes and eosinophils during the ten-day period (Two-way ANOVA, P<0.05). Additional statistical analysis revealed an elevated number of both neutrophils and macrophages at day 2 and day 5 in the methimazole group compared to the vehicle group (Fig. 1). These findings indicated that there was an infiltration of immune cells that are likely involved in phagocytosis, degradation and/or clearance of the damaged OE in the methimazole treatment group.

**Fig 2:**
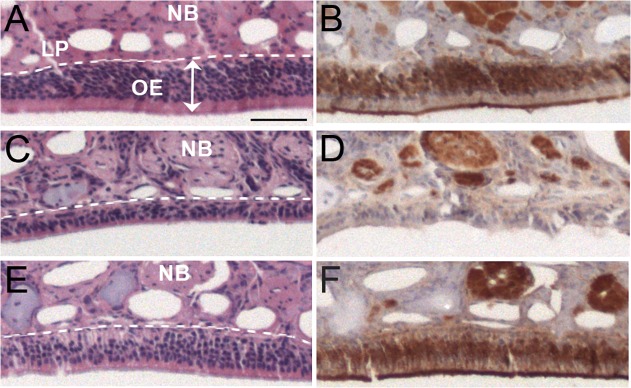
Nasotoxic treatment causes acute loss of the olfactory epithelium followed by regeneration. Syrian hamsters were treated with vehicle (A, B) and methimazole (C to F) by intraperitoneal injection and the olfactory epithelium (OE) was analyzed by histology (e.g., hematoxylin and eosin stain) and for the presence of mature olfactory sensory neurons (e.g., immunohistochemistry for olfactory marker protein) at 11 days (A to D) and 65 days (E, F) after treatment. OSN somata and dendrites are tightly packed in the OE layer while the axons coalesce into nerve bundles in the lamina propria. OMP is normally found in mature OSN, but not in immature OSNs. Thinning of the OE layer is apparent at 11 days after methimazole treatment and few OMP-positive neurons were present in the OE. By 65 days after nasotoxic injury the thickness of the OE is partially restored and regeneration of the OE was evident by the presence of OMP-positive neurons. Lamina propria, LP; nerve bundle, NB; white dotted line is the boundary between the OE and LP. Scale bar is 50 μm.

To monitor the loss and recovery of the OE following methimazole treatment, at day 11 and day 65 post-treatment the morphology of the OE in hamsters was monitored for thickness by hematoxylin and eosin staining, and for mature OSNs by measuring olfactory marker protein, which is expressed in the somata, dendrites, and axons (Fig. 2). At day 11 after methimazole treatment the OE was reduced in size and there was a reduction in OMP expression in the OE, but OMP-positive nerve bundles were still present in the lamina propria (compare the thickness of the OE in Fig. 2A and 2B to Fig. 2C and 2D). Based on prior characterization of the OE after methimazole treatment, the thin cell layer of the OE likely represents early regeneration due to proliferation of neural progenitor cells in the basal layer [25,26]. Over the next two months there is regeneration of functional, mature OSNs as evidenced by OMP-positive cells in the OE, and the OE is restored to its normal thickness (Fig. 2E and 2F).

### The effect of OE loss on prion neuroinvasion by a neurotropic prion strain

**Fig 3:**
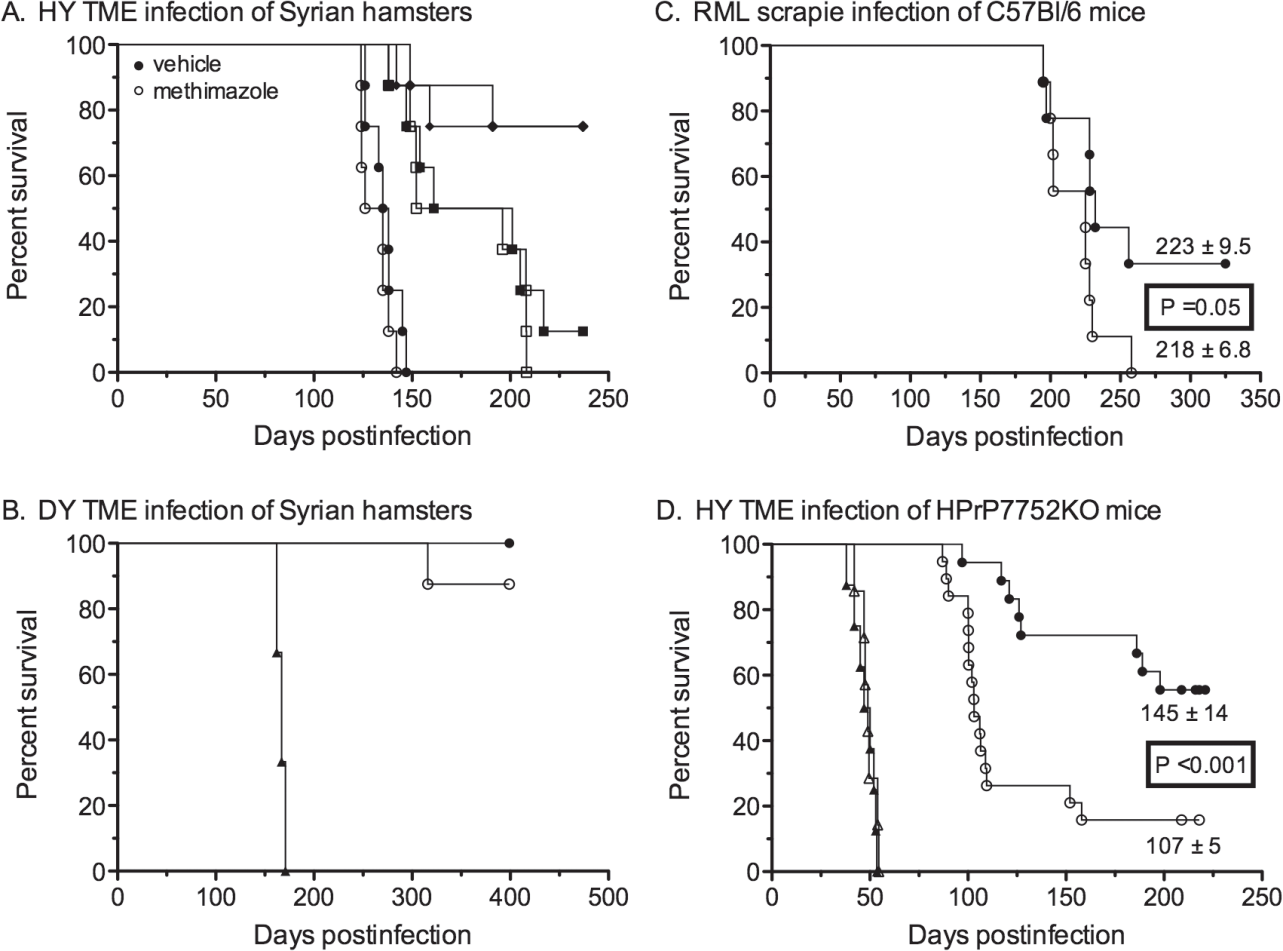
Survival curves following intranasal inoculation of prions in the absence and presence of a preexisting lesion to the olfactory epithelium. Syrian golden hamsters (A, B), wild type C57Bl/6 mice (C), and HPrP7752KO transgenic mice (D) were intranasally inoculated with HY TME (A, D), DY TME (B), and RML scrapie (C). Rodents were pretreated with vehicle (closed symbols) or methimazole (open symbols) two days prior to prion inoculation. (A) Hamsters were inoculated with three doses of serial 10-fold dilutions of HY TME (○,□,◇until disease penetrance was less than 100%. (B, D) Control groups were intracerebrally inoculated with prions (▲,△). Mantel-Cox log-rank test was used to compare the survival curves between vehicle and methimazole treated groups for each route of inoculation and prion dose.

At 48 hours after intraperitoneal injection of Syrian hamsters with vehicle and methimazole either the HY TME or DY TME strain was intranasally inoculated into Syrian golden hamsters. The HY TME strain is both lymphotropic and neurotropic, while the DY TME strain is neurotropic and can only induce disease when it directly enters the nervous system [11,12,40]. HY TME was inoculated at three different doses, 10^4.8^, 10^5.8^, and 10^6.8^ median lethal doses. There was no statistical difference in survival curves and incubation periods between the vehicle and methimazole treated groups (P>0.05). For the two highest doses of inoculum the mean incubation period for the vehicle group were 137± 3.3 and 175 ± 11.3 days compared to 131 ± 2.6 and 176 ± 11.0 days for the methimazole group, respectively (Fig. 3A; ●,○ and ■,□). At the lowest dose of HY TME the mean incubation for the vehicle group was 151 ± 4.3 compared to 170 ± 10.5 for the methimazole group, but there was low disease penetrance at this dose (Fig. 3A;◆,◇). Pretreatment with methimazole also did not have an effect following intracerebral inoculation of Syrian golden hamsters with HY TME (10^4.8^ median lethal doses). The survival curves and mean incubation periods of the vehicle and methimazole treated groups were not different, 78 ± 4.8 days versus 78 ± 4.6 days, respectively (P>0.05). These findings indicate that methimazole disruption of the OE does not alter the disease progression of HY TME following intranasal and intracerebral inoculation.

**Fig 4:**
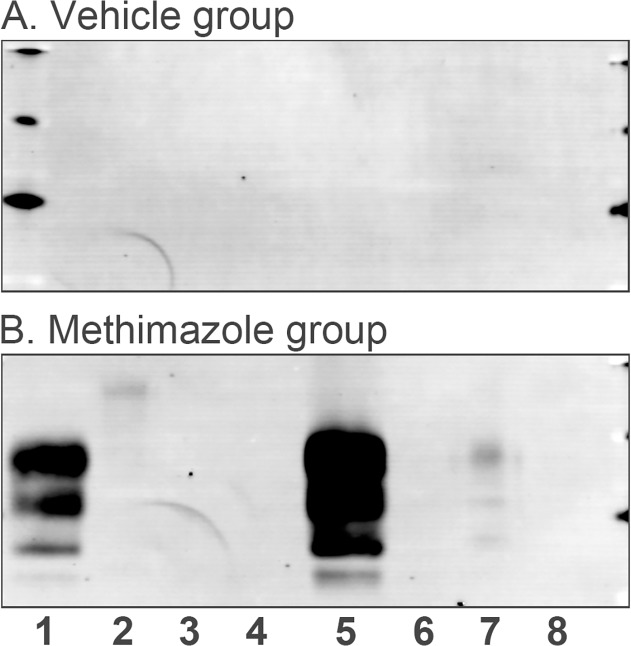
Western blot of prion protein in brain of hamsters following intranasal inoculation of DY TME agent in the absence and presence of a preexisting lesion to the olfactory epithelium. Brain from hamsters at sacrifice (see Table 1) from vehicle (A) and methimazole (B) treatment groups followed by intranasal inoculation of DY TME were enriched for PrP^Sc^ by detergent extraction, differential ultracentrifugation, and proteinase K digestion. For each sample 20 mg tissue equivalents was examined by Western blot for PrP^Sc^, except for two samples in which only 2 mg tissue equivalents was used (B, lanes 1 and 5). None of the hamsters in the vehicle group and only one hamster in the methimazole group exhibited symptoms of DY TME (B, lane 5 and Table 1). The hamster exhibiting clinical symptoms had a strong PrP^Sc^ signal in brain, and two additional hamsters in the methimazole group that were clinically normal and sacrificed after 414 days postinoculation also had evidence of PrP^Sc^ in brain (B, lanes 1 and 7). Molecular weight markers at edge of western blots correspond to 20, 30, and 40 kilodaltons.

A single high dose of DY TME was administered to Syrian golden hamsters because it was previously demonstrated that hamsters are not susceptible to DY TME by the intranasal route [12]. In the vehicle group none of the hamsters exhibited symptoms of disease up to 414 days postinoculation (Fig. 3B; ●). In the methimazole group, one of six hamsters exhibited clinical symptoms at 316 days postinoculation (Fig. 3B; ○). Western blot for PrP^Sc^ in brain was used to confirm that this hamster had developed DY TME infection (Fig. 4 and Table 1). In addition, two of the remaining five hamsters that did not exhibit clinical symptoms by 414 days after methimazole treatment also had evidence of PrP^Sc^ in the brain. One hamster (Fig. 4B, lane 1) had a strong PrP^Sc^ signal that was comparable to the hamster that developed clinical DY TME (Fig. 4B, lane 5), while an additional hamster (Fig. 4B, lane 7) had a weak PrP^Sc^ signal in the brain. None of the asymptomatic vehicle treated hamsters at 414 days postinoculation (N = 4) nor hamsters from either treatment group that died from intercurrent illness between 321 and 365 days (N = 4) had evidence of PrP^Sc^ in the brain (Fig. 4 and Table 1). These findings indicate that disruption of the OE increases the susceptibility to DY TME infection by the nasal route, possibly by establishing direct infection of peripheral nerves.

**Table 1:** Incubation period & PrP^Sc^ status following intranasal inoculation of DY TME in the absence and presence of nasotoxic lesion.

	PrP^Sc^ brain negative		PrP^Sc^ brain positive	
Treatment	Days postinfection	N	Days postinfection	N
Vehicle	>414	4		
	(358)	1		
	(365)	1		
Methimazole	>414	3	316^a^	1
	(321)	1	>414	2
	(326)	1		

^a^ Hamster with clinical symptoms of DY TME.

Numbers in parenthesis are age in days at time of intercurrent death.

N = number of hamsters

Incubation period is 167 ± 2.6 days following intracerebral inoculation of DY TME (Fig. 3B).

### The effect of OE loss on prion neuroinvasion in mice with neuron-restricted prion replication

**Fig 5:**
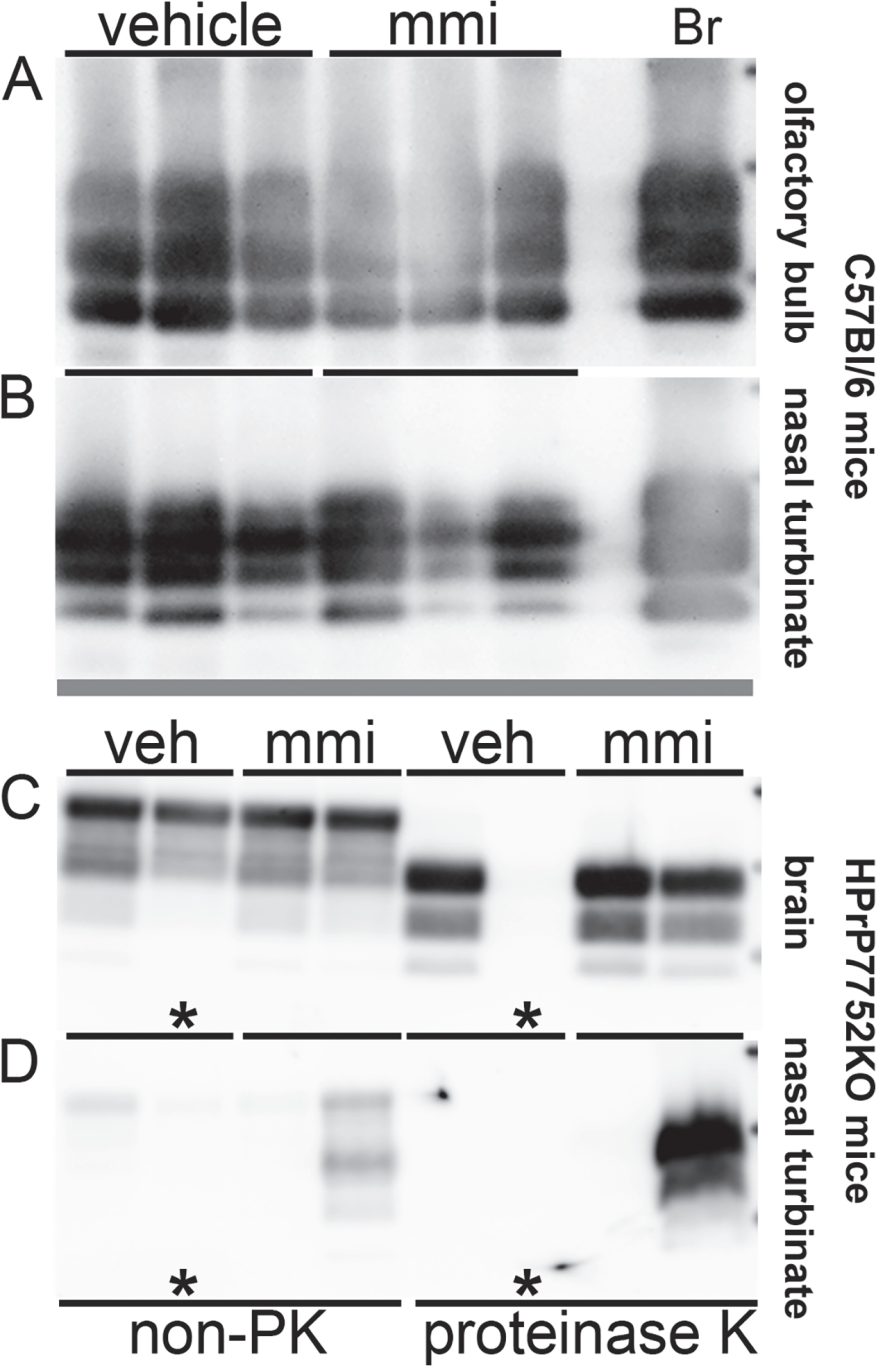
Western blot of prion protein in brain, olfactory bulb, and nasal turbinate of mice following intranasal inoculation of prions in the absence and presence of a preexisting lesion to the olfactory epithelium. Immunodetection of the prion protein in tissues from C57Bl/6 mice (A, B) and HPrP7752KO transgenic mice (C, D) following intranasal inoculation of RML scrapie and HY TME, respectively. Mice were pretreated with vehicle (veh) and methimazole (mmi) 48 hours prior to prion inoculation. Clinically ill C57Bl/6 mice all had PrP^Sc^ deposition in brain (not shown), olfactory bulb (A), and nasal turbinate (B) following proteinase K (PK) digestion of tissue homogenates (A) and PrP^Sc^ enrichment methods that included a PK digestion step (B). In clinically ill HPrP7752KO mice, PrP^Sc^ was detected in brain (C), olfactory bulb (not shown), and in >75% of nasal turbinates (D) following PrP^Sc^ enrichment. Asterisk (C, D) indicates a mouse that did not develop clinical symptoms of prion disease and was devoid of PrP^Sc^. In non-proteinase K (non-PK) treated samples, 20 μg protein from brain (C) and 40 μg protein from nasal turbinate (D) were analyzed while for PK treated samples, 100 μg protein from brain (C) and 1 mg protein from nasal turbinate (D) were used as starting material for PrP^Sc^ enrichment and analysis. RML scrapie-infected brain (Br) control is indicated.

Prion neuroinvasion was also examined after disruption of the olfactory mucosa in wild type C57Bl/6 mice. Following intranasal inoculation of RML scrapie there was no statistical difference in survival curves and incubation periods between the vehicle and methimazole treated groups (223 ± 9.5 days versus 218 ± 6.8 days, P = 0.05) (Fig. 3C; ●,○). All clinical mice in both treatment groups had PrP^Sc^ deposition in the brain, olfactory bulb, and nasal turbinates by western blot (Fig. 5A, 5B, and data not shown). Of the three mice in the vehicle group that did not develop scrapie after 325 days, none had evidence of PrP^Sc^ in the brain by western blot. These results indicate that disruption of the OE did not alter susceptibility of wild type mice to scrapie following intranasal exposure.

Next we examined HPrP7752KO transgenic mice that lack the endogenous murine prion protein gene (*Prnp*), but express the Syrian golden hamster *Prnp* gene under control of the rat neuron-specific enolase promoter [27,28]. These transgenic mice should only enable prion replication in neurons and a subset of neural-derived cell types. Intracerebral inoculation of HPrP7752KO mice with HY TME indicated that pretreatment with methimazole had no effect on survival and incubation period compared to the vehicle group (49 ± 1.5 days versus 48 ± 2.0 days, P>0.05) (Fig. 3D; ▲,△). Following intranasal inoculation, the survival curves for the two groups were statistically different (P<0.001) (Fig. 3D; ●,○). Furthermore, the vehicle group had a mean incubation period of 145 ± 14 days (8 of 19 mice affected) while the methimazole group had a mean incubation period of 107 ± 5 days (16 of 19 mice affected) indicating that there was a >25% reduction in the mean incubation period in the methimazole group. Of the clinical mice in both treatment groups, PrP^Sc^ was detected by western blot in the brain (21 of 21 mice), olfactory bulb (17 of 18 mice), and nasal turbinate (14 of 18 mice) (Fig. 5C and data not shown; not all tissues were available for analysis). Of the eleven mice in the vehicle group and three in the methimazole group that did not exhibit clinical symptoms by 210 days postinoculation, none had evidence of PrP^Sc^ deposition in the brain by western blot (Fig. 5C asterisks and data not shown). These findings indicate that when *Prnp* expression is restricted to neurons and there is a disruption of the OE then there was an increase in susceptibility to prion infection and a dramatic shortening of the incubation period following intranasal inoculation.

### Temporal and spatial analysis of prion neuroinvasion following OE loss

**Fig 6:**
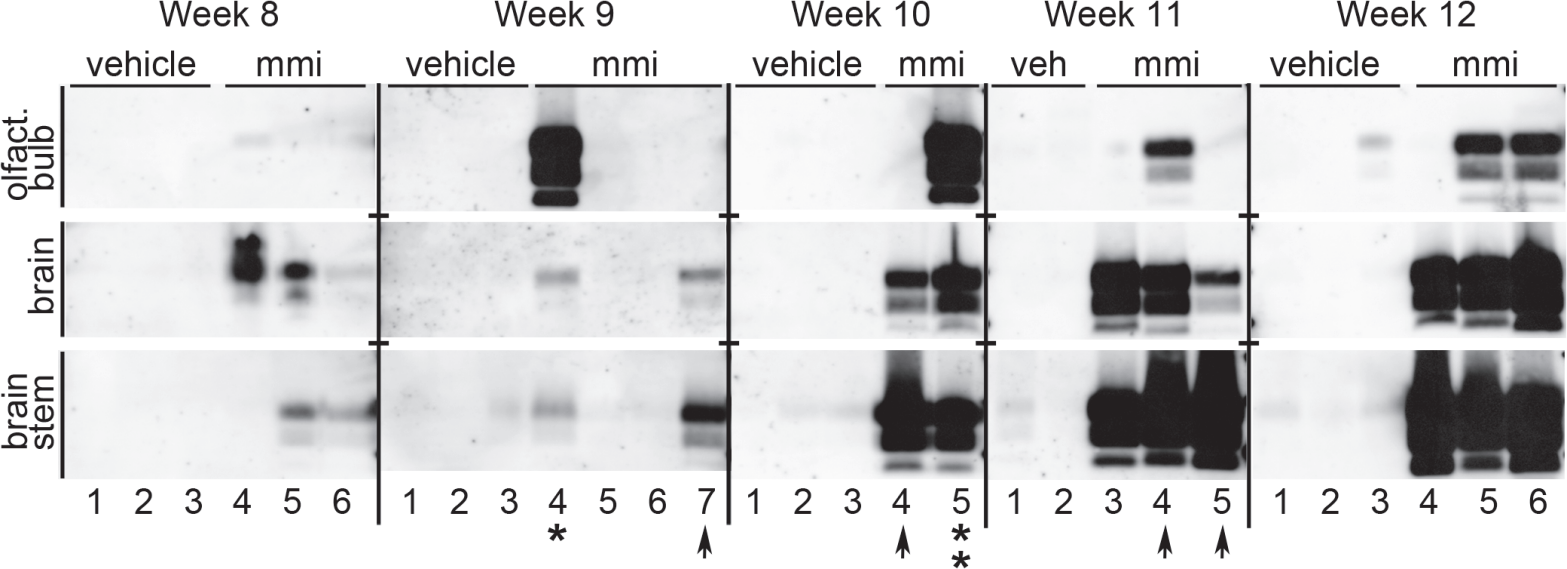
Temporal and spatial analysis of PrP^Sc^ deposition in the brain, brain stem, and olfactory bulb following intranasal inoculation of HY TME in the absence and presence of a preexisting lesion to the olfactory epithelium. HPrP7752KO mice were treated with vehicle (veh) or methimazole (mmi) 48 hours prior to intranasal inoculation of HY TME. Two to four mice from each treatment group were collected weekly between 8 and 12 weeks postinoculation and the brain, brain stem, and olfactory bulb were dissected for PrP^Sc^ analysis by western blot. Asterisks indicate mice with PrP^Sc^ deposition pattern suggestive of initial prion infection of olfactory bulb, while arrowheads are indicative of initial prion infection of brain stem.

Prior studies in wild type rodents indicate that following intranasal inoculation prions can enter the brain by non-olfactory neural routes [41] and we were interested to determine if the acceleration of prion disease from the nasal route in HPrP7752KO mice treated with methimazole can affect the route of prion entry into the brain. For these studies HPrP7752KO mice were divided into vehicle and methimazole groups and were examined weekly for PrP^Sc^ deposition between eight and twelve weeks postinoculation of HY TME and prior to the onset of clinical symptoms. In the vehicle group one or two samples in the brain stem were weakly positive for PrP^Sc^ at each time point from nine to twelve weeks postinoculation (Fig. 6). However, there was not a noticeable increase in PrP^Sc^ levels in the brain stem over this time span, which would be expected if scrapie had entered the central nervous system and began to replicate. This lack of progression of prion infection in the brain stem was confirmed by an absence of PrP^Sc^ in brain from the vehicle group between week 8 and 12 postinoculation (Fig. 6). Only a single olfactory bulb, in animal #3 at week 12, was weakly positive in the vehicle group suggesting neuroinvasion may involve the olfactory nerve since PrP^Sc^ was not detected in the brain of this mouse. The lack of an increase in PrP^Sc^ levels in the brain stem and the absence of PrP^Sc^ in the brain in the vehicle group suggest that prion neuroinvasion is not a prominent feature prior to 12 weeks postinoculation.

In contrast, in the methimazole group at week 8 and 9 postinoculation there was weak to moderate levels of PrP^Sc^ in both the brain stem and brain. At least one brain region in five of seven mice had evidence of PrP^Sc^ at these early time points (Fig. 6). In the olfactory bulb, at least two animals had evidence for weak PrP^Sc^ deposition at week 8 postinoculation. At week 9 there was a very high level of PrP^Sc^ in the olfactory bulb of animal #4 (Fig. 6, asterisk), which was considerably greater than in the brain and brain stem suggesting that in this animal HY TME entered the brain via the olfactory bulb prior to spreading to other regions of the CNS. In contrast, in animal #7 at week 9 there is a greater level of PrP^Sc^ in the brain stem compared to the brain while a signal was not observed in the olfactory bulb (Fig. 6, arrowhead). This finding suggests that prions initially entered into the brain stem prior to spread to the brain in this mouse.

In weeks 10 through 12 postinfection there was a steady increase and moderate-to-strong PrP^Sc^ deposition pattern in both the brain stem and brain in all animals at each time point in the methimazole group. At week 10 and 11 there were several animals that had a strong PrP^Sc^ deposition in the brain stem and either an absence of PrP^Sc^ or low-to-moderate PrP^Sc^ levels in the brain and olfactory bulb (Fig. 6, arrowheads). These findings also suggest that there is an earlier onset of HY TME agent entry and replication in the brain stem compared to the brain and olfactory bulb. In addition, four of eight mice had evidence of PrP^Sc^ deposition in the olfactory bulb from weeks 10 through 12 postinoculation. Of these HPrP7752KO mice one had an intense PrP^Sc^ signal in the olfactory bulb that was higher than that found in the brain and brain stem suggesting initial entry into the olfactory bulb prior to other regions of the brain (Fig. 6, week 10 animal #5, double asterisk). In other animals the PrP^Sc^ distribution did not reveal a pattern of entry or temporal spread within the brain. Overall, these findings suggest that following disruption of the OE that the brain stem is the earliest site of prion accumulation prior to spread to other brain regions in some animals, while in other HPrP7752KO mice the olfactory bulb appears to be the initial site of prion replication.

**Fig 7:**
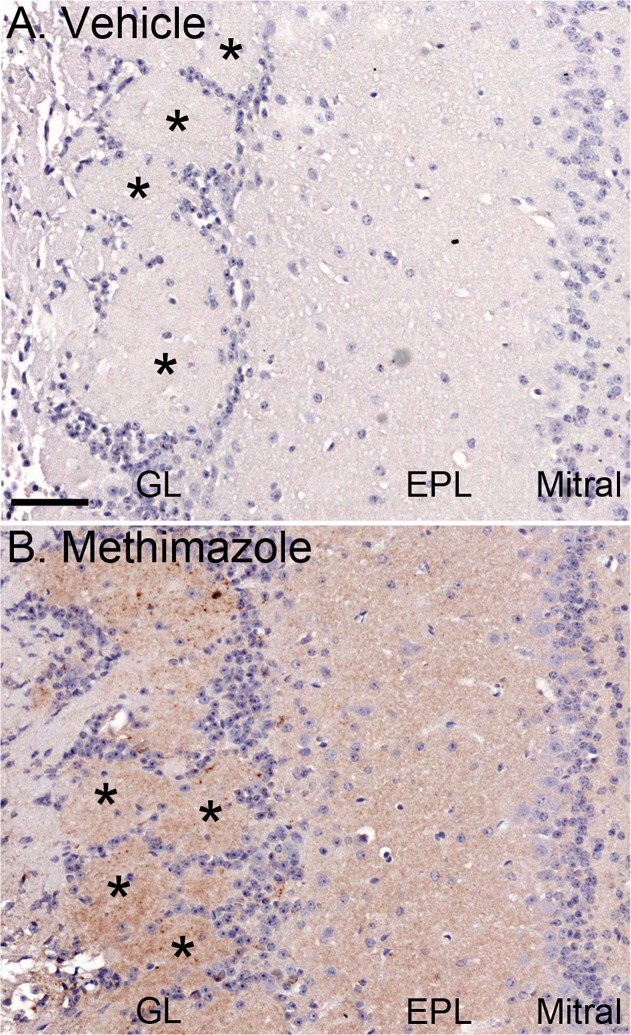
PrP^Sc^ deposition in the olfactory bulb of HPrP7752KO mice at eight weeks after intranasal inoculation with HY TME in the absence and presence of a preexisting lesion to the olfactory epithelium. HPrP7752KO mice were treated with vehicle (A) or methimazole (B) at 48 hours prior to intranasal inoculation of HY TME. At eight weeks after infection, immunohistochemistry on the olfactory bulb illustrates PrP^Sc^ deposition (reddish brown color) in glomeruli (*) in the glomerular layer (GL), the external plexiform layer (EPL), and the mitral cell layer in methimazole, but not vehicle, treated transgenic mice. Tissue sections were counterstained with hematoxylin. Scale bar is 25 μm.

PrP^Sc^ immunohistochemistry was also used to analyze prion infection in the brain of HPrP7752KO mice at early time points before widespread dissemination of HY TME infection. In the vehicle treatment group, PrP^Sc^ deposition was not observed in the brain (N = 14), brain stem (N = 14), and olfactory bulb (N = 13) between 8 and 12 weeks postinfection (Fig. 7A). In the methimazole group, PrP^Sc^ deposition was found in the brain stem (3 of 14 mice) and olfactory bulb (3 of 15 mice), but not in the brain (N = 14) of HPrP7752KO mice. In the three PrP^Sc^-positive mice for each brain region, PrP^Sc^ was observed in the olfactory bulb in one mouse at 8 weeks and in two mice at 12 weeks postinfection; in the brain stem PrP^Sc^ was found in one mouse at 10 weeks and in two mice at 12 weeks postinfection. The PrP^Sc^ staining intensity was stronger in the olfactory bulb, but overall, there were a lower number of PrP^Sc^-positive mice detected using immunohistochemistry compared to western blot. The PrP^Sc^ deposition pattern was similar in the olfactory bulbs at these early time points and consisted of multiple, small PrP^Sc^ aggregates located in glomeruli, which are synapse-rich structures containing OSN nerve terminals and dendrites of olfactory bulb neurons (Fig. 7B). There was additional PrP^Sc^ deposition in the external plexiform layer, and less often in the mitral cell layer and inner plexiform layer. These findings were consistent with PrP^Sc^ tissue analysis by western blot and they also suggest prion entry into the olfactory bulb and brain stem following lesion of the olfactory mucosa. These results also support a delay, or reduced level, of prion entry into the brain in the vehicle treatment group following HY TME infection.

## Discussion

In the current study we demonstrate that nasotoxic treatment, which causes an acute loss of the OE, inflammation in the nasal airway, and regeneration of OSNs, can increase susceptibility to prion infection and shorten the disease course in rodent models in which prion replication is restricted to neurons. This accelerated onset of prion disease is associated with earlier prion neuroinvasion into the olfactory bulb and brain stem. In natural prion diseases exposure of mucosae carries the highest risk of prion entry and establishment of infection in the regional LRS prior to spread into the nervous system. In mucosa containing a high density of neurons, such as the olfactory epithelium, injury to the epithelium may increase access to the nervous system and provide a direct route of prion neuroinvasion. The current study suggests that disruption of the integrity of the OE, which is not uncommon in ruminants, may enhance prion neuroinvasion from the olfactory nerve and brain stem associated cranial nerves that innervate the nasal mucosa. This conclusion is consistent with the unique features of OSNs, including 1) they are exposed to the environment in the nasal airway and project to the olfactory bulb; and 2) they are susceptible to damage and death by environmental toxins, chemicals, and microorganisms as well as by inflammation and allergic responses [16–24]. The OSNs are also a site of prion infection [12,32,41–46] and moderate levels of prion infectivity can be released into nasal secretions [31,32,47]. A recent study demonstrates that diagnosis of Creutzfeldt-Jakob disease in humans can be made from a brushing of the OE [48].

Our findings also indicate that nasotoxic treatment does not alter the prion incubation period in rodent models that are permissive for prion replication in the LRS, which is in contrast to animal models where prion replication is restricted to neurons. An explanation that may reconcile these results is that the prion incubation period is not significantly different when prion replication in the LRS is followed by subsequent neuroinvasion, versus direct infection of the peripheral nervous system following nasotoxic lesion. In RML-infected wild type mice and HY TME-infected hamsters, nasotoxic lesion may only result in a slight enhancement in neuroinvasion from the olfactory mucosa, but to a degree that does not alter the incubation period compared to the LRS-dependent pathway. However, in neuron-restricted models of prion replication mucosal exposure may be an inefficient route to establish prion infection in the absence of a LRS replication phase, but in these models nasotoxic lesion can enhance prion neuroinvasion from the OE. There is some evidence to support the explanation that a peripheral and central route of inoculation can result in a similar onset of clinical disease in ruminants. In experimental infection of mule deer and white-tailed deer with chronic wasting disease similar or overlapping incubation periods were observed following per os, aerosolization, and intracerebral inoculations [49–52]. It should be noted that the variance in the length of the prion incubation period increases as the prion dose decreases, so overlapping incubation periods upon natural exposure to low doses of prions may obscure the distinction between peripheral and central routes of infection [53,54]. In contrast to CWD, experimental infection of sheep with scrapie by peripheral routes of exposure typically have longer incubation periods than intracerebral inoculation [55–57]. However, there is one report that the incubation periods were similar following intralingual and intracerebral inoculation of scrapie [58]. Studies in rodents indicate that intralingual inoculation leads to rapid neuroinvasion via cranial nerves [12,14]. In rodent models in which the source and dose of the prion inoculum, and the host prion protein genotype are known, peripheral exposure to prions results in longer incubation periods than direct infection of the nervous system [14,59,60]. Although exposure that leads to direct entry of prions into the peripheral nervous system results in shorter incubation periods than peripheral routes that involve LRS-dependent neuroinvasion [11,12,14,61]. A reason for these discrepancies among prion diseases may be due to differences among host species in permissiveness and/or efficiency to establish prion infection in the LRS, and the initial steps in neuroinvasion, which for the LRS is defined by the ability to transfer prion infection from the LRS to the nerves that innervate lymphoid tissue [62,63].

Several scenarios can explain the accelerated prion neuroinvasion and disease onset following lesion of the olfactory mucosa. These include direct entry into nerve terminals of the trigeminal ganglia and/or uptake by the perineurium surrounding the nerve bundles of the OSNs that remain within the lamina propria, which may become more accessible to infection after injury to the OE. The perineurium of olfactory nerve bundles has a role in drainage of the cerebrospinal fluid (CSF) into the nasal lymphatics [64] and is also a site of PrP^Sc^ deposition in sheep scrapie [46]. Methimazole-induced fragmentation and loss of the OE may compromise the CSF-lymphatic system pathway as well as expose OSN dendritic knobs, dendrites, somata, and axons that may be protected from direct prion infection when the mucus layer of the OE is intact [31]. In the neuron-restricted models of prion replication, direct infection of neurons in the OE could explain the earlier onset of neuroinvasion and shorter incubation period in HY TME-infected HPrP7752KO mice and the increase in prion neuroinvasion in DY TME infection of hamsters in the methimazole group. In the absence of LRS infection, greater access to the cranial nerves in the olfactory mucosa would be expected to result in enhanced prion neuroinvasion. A second explanation for accelerated prion neuroinvasion and disease onset following nasotoxic lesion is establishment of prion infection in neural progenitor cells and immature neurons in the OE. The neural progenitor cells in the basal layer of the OE undergo increased cell division and neurogenesis in order to replace the OSNs lost after methimazole treatment [25,65–68]. Both neural progenitor cells and immature OSNs express the cellular form of the prion protein, PrP^C^ [69,70], and therefore, are potential cellular sites for prion replication since mature OSNs can replicate prions to high levels [31,32,44,45,71]. If HY TME infection in HPrP7752KO mice and DY TME infection in hamsters can be established in immature OSNs following methimazole treatment, then we would predict that as these neurons mature and integrate into the neural circuitry that this would lead to infection of the olfactory nerve and the OSN axon terminals in the glomeruli of the olfactory bulb. Subsequent transynaptic spread of prions from OSN nerve terminals to olfactory bulb neurons that synapse at the glomeruli could transfer infection into the brain. Further studies are needed to dissect these pathways, but it is intriguing to speculate that neural progenitor cells or neural stem cells are potential targets of prion infection.
